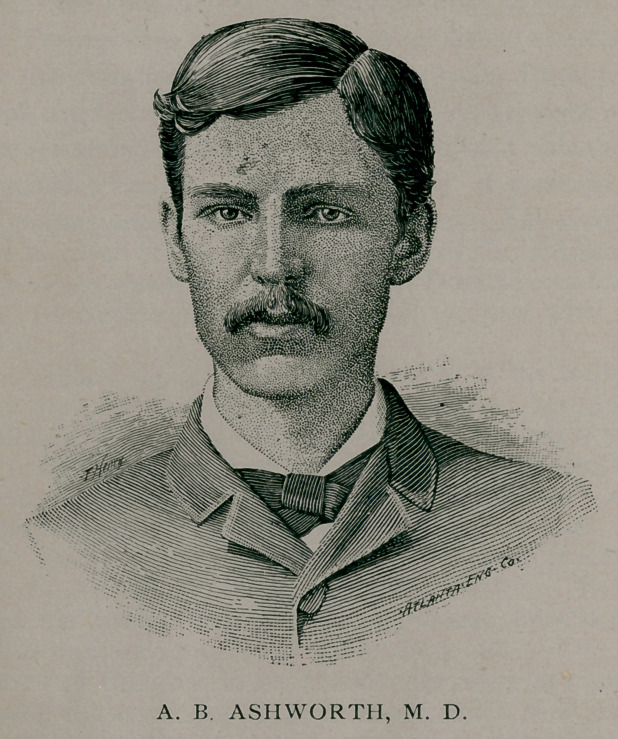# Dr. A. B. Ashworth

**Published:** 1889-06

**Authors:** 


### Editorial.

### DR. A. B. ASHWORTH.

It is with deep sorrow that we have to announce to our readers the death of Dr. A. B. Ashworth, the business manager of the JOURNAL, which occurred suddenly in this city on the 18th inst. The long association of the deceased with the business interests of the JOURNAL will doubtless make the announcement a sad one to all who had been brought into relation with him. For several years he had occupied a position in the editorial office, first as assistant, and after the death of Dr. Gray as sole manager, and a considerable part of the success to which the JOURNAL has attained has been due to the faithful performance of his duties in both capacities.

Dr. Ashworth was a graduate of the Atlanta Medical College in the class of 1886. After his graduation he decided not to enter upon the practice of his profession, but to devote his energies and his fine business talents to the interest of the ATLANTA MEDICAL AND SURGICAL JOURNAL. To this decision he was led partly by his strong friendship for Dr. Gray and partly by his tastes and business talents, by which he was well fitted for this class of work. The success which has attended his labors in this direction attests the wisdom of his decision.

He was a member of the Medical Association of Georgia, and in 1887 was appointed to fill the vacancy in the office of Secretary of the Association, caused by the death of Dr. Gray. Upon his retirement from this office the Association voted him a gratuity of one hundred dollars as an evidence of its appreciation of his services. He was also an honored member of the Atlanta Society of Medicine and took great interest in its welfare and success, although circumstances prevented a frequent attendance upon its meetings.

He was steadfast in his friendship, honorable in all his transactions, and generous to a fault. Although quiet and unostentatious in his manners, he possessed a capacity for dealing with men and affairs, which contributed largely to his success in the laborious field of medical journalism. Although a young man he successfully carried on a work which had failed in the hands of men of maturer years and larger experience.

The death of Dr. Ashworth is a serious and unexpected blow to the JOURNAL; yet, the work which has fallen from his hands will be taken up and carried on by others, and no effort will be spared to maintain the high position to which it has been carried by him. Arrangements have already been made for the continuance of its publication under the same editorial management as heretofore, and we can assure our subscribers and advertisers that they will not be allowed to suffer from the loss which has fallen upon us, as Dr. Asworth made express provision in his will for the prompt and uninterrupted appearance of the JOURNAL in case of his death. Respect for the memory of a useful member of our staff, and of the good which he has wrought, will furnish an incentive to renewed energies in preserving unimpaired the valuable results of his labors.

A. B. ASHWORTH, M. D.

## Figures and Tables

**Figure f1:**